# Cyclin B1–Cdk1 Activation Continues after Centrosome Separation to Control Mitotic Progression

**DOI:** 10.1371/journal.pbio.0050123

**Published:** 2007-05-01

**Authors:** Arne Lindqvist, Wouter van Zon, Christina Karlsson Rosenthal, Rob M. F Wolthuis

**Affiliations:** 1 Department of Cell and Molecular Biology, Karolinska Institutet, Stockholm, Sweden; 2 Division of Molecular Biology/P2, The Netherlands Cancer Institute, Amsterdam, The Netherlands; Dana-Farber Cancer Institute, United States of America

## Abstract

Activation of cyclin B1–cyclin-dependent kinase 1 (Cdk1), triggered by a positive feedback loop at the end of G2, is the key event that initiates mitotic entry. In metaphase, anaphase-promoting complex/cyclosome–dependent destruction of cyclin B1 inactivates Cdk1 again, allowing mitotic exit and cell division. Several models describe Cdk1 activation kinetics in mitosis, but experimental data on how the activation proceeds in mitotic cells have largely been lacking. We use a novel approach to determine the temporal development of cyclin B1–Cdk1 activity in single cells. By quantifying both dephosphorylation of Cdk1 and phosphorylation of the Cdk1 target anaphase-promoting complex/cyclosome 3, we disclose how cyclin B1–Cdk1 continues to be activated after centrosome separation. Importantly, we discovered that cytoplasmic cyclin B1–Cdk1 activity can be maintained even when cyclin B1 translocates to the nucleus in prophase. These experimental data are fitted into a model describing cyclin B1–Cdk1 activation in human cells, revealing a striking resemblance to a bistable circuit. In line with the observed kinetics, cyclin B1–Cdk1 levels required to enter mitosis are lower than the amount of cyclin B1–Cdk1 needed for mitotic progression. We propose that gradually increasing cyclin B1–Cdk1 activity after centrosome separation is critical to coordinate mitotic progression.

## Author Summary

When active, the enzyme cyclin B1–cyclin-dependent kinase 1 (Cdk1) commits a growing cell to the process of mitotic cell division and chromosome separation. Cyclin B1–Cdk1 activation is controlled in many ways, but once its activity rises above a certain level, further activation of cyclin B1–Cdk1 is catalyzed by a positive-feedback loop. This generates highly active cyclin B1–Cdk1 and triggers the start of mitosis, which can only be completed when cyclin B1–Cdk1 activity is properly shut off again. However, it is not clear how cyclin B1–Cdk1 activity develops in human cells or how the switch between its inactive and active states is controlled. Our work combines activation measurements with a kinetic model to study how cyclin B1–Cdk1 activity accumulates just before and during mitosis. We show that cyclin B1–Cdk1 activity develops gradually in early mitosis and that different activity levels are required for initiation of, and progression through, mitosis. We also demonstrate that once cyclin B1–Cdk1 activation is truly launched, it is bound to continue and will not lightly drop back again. We propose that the successive cyclin B1–Cdk1 activity levels by themselves may coordinate the progression through the distinct phases of mitosis.

## Abstract 3

The gradual increase of cyclin B1-Cdk1 activation in human cells is proposed to be critical for the progression of mitosis.

## Introduction

Mitotic entry is catalyzed by the kinase activity of cyclin-dependent kinase 1 (Cdk1) in complex with cyclin B1 [1]. Cyclin B1 levels first rise during G2 phase, which allows the accumulation of cyclin B1–Cdk1 complexes [2]. At this stage, cyclin B1–bound Cdk1 is kept inactive by phosphorylation of T14 and Y15 by Wee and Myt kinases [3,4]. Subsequently, cyclin B1–Cdk1 can be activated by Cdc25 phosphatases [5]. They dephosphorylate Cdk1 in two separate binding steps, in which T14 is dephosphorylated before Y15 [6,7]. In interphase *Xenopus* egg extracts, cyclin B1–Cdk1 complexes have low activity. Cdk1 activity in cyclin B1 immunoprecipitates rises gradually through G2, eventually reaching approximately 30% of its maximum activity at the end of G2 [8–10]. At this point, a threshold concentration is reached that autocatalyzes rapid further activation of cyclin B1–Cdk1 and triggers entry into mitosis [10].

Cyclin B1–Cdk1 activation contributes to the separation of centrosomes in late G2 [11,12]. In human cells, active cyclin B1–Cdk1 is initially detected on centrosomes, shortly before they start to migrate apart at the end of G2 or in prophase [13,14]. Active cyclin B1–Cdk1 is then phosphorylated on its cytoplasmic retention sequence, leading to nuclear translocation of cyclin B1–Cdk1 and, subsequently, to enhanced chromosome condensation and nuclear envelope breakdown [15,16]. When cells are in mitosis, the paired sister chromatids need to be correctly connected to the mitotic spindle, a process that is governed by the spindle checkpoint. This checkpoint inhibits ubiquitin ligase activity of the anaphase-promoting complex/cyclosome (APC/C), thereby preventing premature cyclin B1 destruction and Cdk1 inactivation. The sustained high cyclin B1–Cdk1 activity allows cells to stay in mitosis as long as is required for all chromosomes to attach to the mitotic spindle in a bioriented fashion [17,18]. After requirements for the checkpoint have been met, progressive loss of cyclin B1–Cdk1 activity is essential for successful chromosome segregation and completion of cell division [17].

It has been proposed that such a cyclin B1–Cdk1–APC/C module could function as an autonomous oscillator governing cell cycle progression [19–21]. Based on the ability of cyclin B1–Cdk1 to inhibit its inhibitor Wee1 [22–25], to activate its activators of the Cdc25 phosphatase family [26,27], and to stimulate cyclin B1 destruction [28], two major theories of mitotic cyclin–cyclin-dependent kinase (Cdk) activation have been proposed (see Text S1 and Figure S1 for additional information on these models). Some models suggest a limit cycle behavior, in which the levels of Cdk activity will never be stable but oscillate between an inactive and an active state [19,29,30]. Other models describe a bistable system, in which thresholds for activation and inactivation differ and Cdk1 is either inactive, active, or approaching one of these stable states [31–33]. One of the differences between these systems is that, in a bistable system, once a threshold level of cyclin B1–Cdk1 complexes is reached and activation has proceeded, cyclin B1–Cdk1 will not be inactivated even though the cyclin B1–Cdk1 levels are reduced to levels below the activation threshold. This feature of a bistable system is referred to as hysteresis. A commitment of cells to start and complete mitosis once they have activated cyclin B1–Cdk1, is strikingly reminiscent of certain features of bistability. Various models and some experimental data indeed point to bistability governing mitotic Cdk1 activation [34–36] (Figure S1). It has also been argued, however, that the experimental set-ups used so far may have created an artificial bistable system [29]. Furthermore, recently, Csikasz-Nagy and coworkers [37] suggested that both bistability and limit cycle oscillations occur, depending on the cellular context.

Although it is clear that cyclin B1–Cdk1 is mostly inactive in G2 and highly active in mitosis, methods to follow the development of cyclin B1–Cdk1 activity during the rapidly successive mitotic stages have been lacking. As a result, experimental data of Cdk1 activation kinetics in human cells are very limited. Such data could, for instance, reveal whether cyclin B1–Cdk1 activation, once initiated, is sufficiently robust to drive mitotic progression even in the cytoplasm, when a large fraction of cyclin B1–Cdk1 translocates to the nucleus in prophase. This would be indicative of a bistable Cdk1 activation response and further indicate that precise regulation of cyclin B1–Cdk1 activity could control mitotic events per se.

We use novel assays to determine how cytoplasmic cyclin B1–Cdk1 activation proceeds in human cells. We find that cyclin B1–Cdk1 is activated in late G2 and that the activity gradually increases between centrosome separation and prometaphase. Moreover, we investigated the effect of partially reducing Cdk1 levels on mitotic progression. We show that whereas low levels of Cdk1 are sufficient for mitotic entry, higher levels are needed for normal mitotic progression and initiation of anaphase. Altogether, we show that the development of cyclin B1–Cdk1 activity in vivo proceeds as if it were governed by bistability. Our results explain how cyclin B1–Cdk1 can be responsible for cytoplasmic rearrangements even during nuclear translocation. We propose a model in which different thresholds of cyclin B1–Cdk1 activity, in combination with a gradual increase in activity, help to coordinate early and late mitotic events.

## Results/Discussion

### Temporal Development of Cyclin B1–Cdk1 Activity in Single Cells

Cyclin B1–Cdk1 is initially activated on centrosomes in late G2, shortly before they start to migrate apart [13,14]. To determine how cyclin B1–Cdk1 activation proceeds, we developed an assay to follow the pattern of cyclin B1–Cdk1 activity in single human cells. Because Cdk1 levels are in excess of cyclin levels in mammalian cells [38] (unpublished data) and because Cdk1 is phosphorylated only when in complex with a cyclin [10], we reasoned that the ratio between phosphorylated Cdk1 and cyclin B1 could provide an estimate of the relative activity of the cyclin B1–Cdk1 complex. Therefore, we used an antibody recognizing Cdk1 when phosphorylated on Y15 (Cdk1 phosphorylation [Cdk1-P], which represents inactive Cdk1) combined with an antibody recognizing cyclin B1, in immunofluorescence experiments.

We first wanted to test the specificity of these antibodies, and therefore we used short hairpin RNA (shRNA) to reduce the levels of cyclin B1 or Cdk1 in cells. The cytoplasmic staining of the Cdk1-P antibody in G2 almost completely disappeared by microinjecting cells with an shRNA to cyclin B1 or Cdk1, showing the cytoplasmic signal represents phosphorylated Cdk1 (Figure S2). However, Cdk1 RNA interference (RNAi) could not totally eliminate the staining in interphase nuclei and on centrosomes, indicating some cross-reactivity in these locations, most probably with phosphorylated Cdk2. Furthermore, the cyclin B1 and Cdk1-P signal colocalized linearly in the cytoplasm, indicating that the signal represents cyclin B1–Cdk1 complexes (Figure S2). Therefore, in this study we exclusively focused on cytoplasmic cyclin B1–Cdk1 activation, which we could monitor specifically.

**Figure 1:**
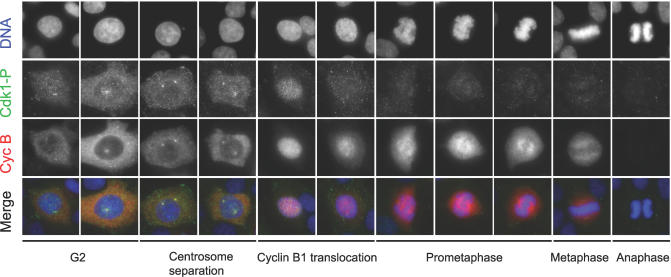
Immunofluorescence Pattern of Cyclin B1 and Y15 Phosphorylated Cdk1 from Early G2 Phase to Anaphase in HeLa Cells Fluorescence images are undeconvolved maximum intensity projections of unsynchronized cells from a single cover slip. Cells are presented from G2 (left) to anaphase (right). Top row: DNA, second row: phosphorylated Cdk1, third row: cyclin B1, bottom row: merge of all three fluorescence channels.

Figure 1 shows maximum intensity projections of cells in various stages of G2 and mitosis, costained for chromosomes, phospho-Cdk1, and cyclin B1. In G2 and mitotic cells, cyclin B1, shown in the second panel, colocalized with cytoplasmic phospho-Cdk1, apparently decreasing around the time when cyclin B1 (third panel) translocated to the nucleus. In metaphase, cyclin B1 levels declined, whereas Cdk1-P appeared to remain similar to the level in prometaphase cells. In anaphase, both cyclin B1 and phospho-Cdk1 levels had largely disappeared.

Subsequently, we analyzed numerous undeconconvolved fluorescent images of G2 and mitotic cells, to carefully quantify the cytoplasmic cyclin B1 and Cdk1-P staining in various phases of G2 and mitosis (see Materials and Methods and Figure S3 for details on image acquisition and data analyses). To control for changes in morphology during mitotic progression, we performed quantitations of nuclear factor κB (NF-κB), a protein that is cytoplasmic in normal cells and has no obvious function in mitosis (Figure S4). We exclusively analyzed endogenous proteins in unsynchronized HeLa cells under normal growth conditions.

**Figure 2:**
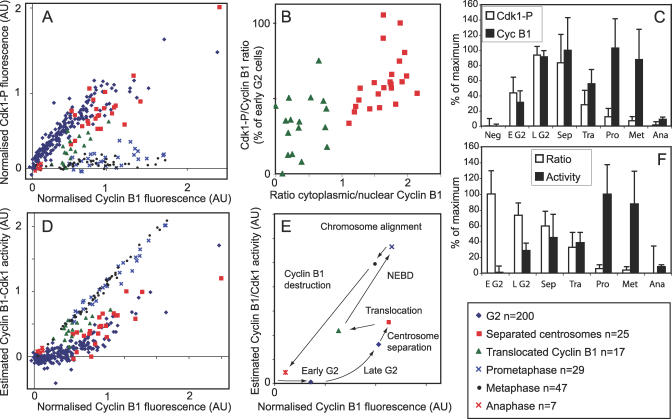
Gradual Dephosphorylation of Cdk1 in Mitotic Entry (A) Quantification of immunofluorescence labeling. Cytoplasmic Cdk1-P (*y*-axis) and cyclin B1 signal (*x*-axis) were quantified in the cytoplasm of unsynchronized cells. Each dot corresponds to one cell. Cells were labeled according to morphology and staining as follows: cells with cytoplasmic cyclin B1 (blue diamonds), cytoplasmic cyclin B1 and separated centrosomes (red squares), predominant cyclin B1 staining in nucleus (green triangles), condensed DNA and no distinguishable nucleus in cyclin B1 staining (light blue Xs), all chromosomes aligned on a metaphase plate (black dots), and anaphase (red Xs). (B) Relative cyclin B1–Cdk1-P during nuclear translocation. Ratio of cytoplasmic Cdk1-P and cyclin B1 signal (*y*-axis) in cells with separated centrosomes (red squares) and translocated cyclin B1 (green triangles) correlated with the ratio of nuclear and cytoplasmic cyclin B1 (*x*-axis). The average cytoplasmic Cdk1-P/cyclin B1 ratio of early G2 cells is defined as 100%. Early G2 cells are defined as cells with cytoplasmic cyclin B1 levels between average anaphase cyclin B1 levels and lowest cyclin B1 level of cell with separated centrosomes. (C) Average values of cytoplasmic cyclin B1 and phosphorylated Cdk1. Values are shown as percentage of the highest average. Error bars indicate SD. Neg, cells used for background subtraction; E G2, G2 cells with cyclin B1 levels between average anaphase levels and lowest value of cell with separated centrosomes; L G2, G2 cells with cyclin B1 levels above lowest value of cell with separated centrosomes; Sep, separated centrosomes; Tra, translocated cyclin B1; Pro, prometaphase; Met, metaphase; Ana, anaphase. (D) Estimated cytoplasmic cyclin B1/Cdk1 activity of individual cells. The degree of dephosphorylation multiplied with the level of cyclin B1 (*y*-axis), as a function of the level of cyclin B1 (*x*-axis). Cells are labeled as in (A). (E) Average values of estimated cytoplasmic cyclin B1–Cdk1 activities within the distinct phases, shown in Figure 3D, as a function of cytoplasmic cyclin B1 levels. (F) Average values of cytoplasmic Cdk1-P/cyclin B1 ratio and estimated cytoplasmic cyclin B1–Cdk1 activities in the different phases. Values are shown as percentage of the highest average. Error bars indicate SD. Labels are as in Figure 2C.

After correction for changes in morphology, we subsequently plotted cytoplasmic Cdk1-P levels as a function of cytoplasmic cyclin B1 levels. We observed that both Cdk1-P and cyclin B1 increased in a roughly linear fashion when cells proceeded through G2, with little change in the Cdk1 phosphorylation (P) state, in agreement with cyclin B1–Cdk1 complexes displaying low activity in early G2 (Figures 1 and 2A; blue diamonds). As we showed previously [14], a subset of cells with nonseparated centrosomes and high cyclin B1 levels contained reduced levels of phosphorylated Cdk1, indicating a pool of cyclin B1–Cdk1 is partially active. In cells with high cyclin B1 levels, we saw Cdk1 dephosphorylation further proceeding when centrosomes started to migrate apart. The dephosphorylation gradually continued until chromosomes were aligning in prometaphase (Figures 1 and 2A; top left, red squares and light-blue Xs), whereas total Cdk1 levels did not decline (Figure S5). This strongly indicates cyclin B1–Cdk1 activation progressively develops during mitosis.

**Figure 3:**
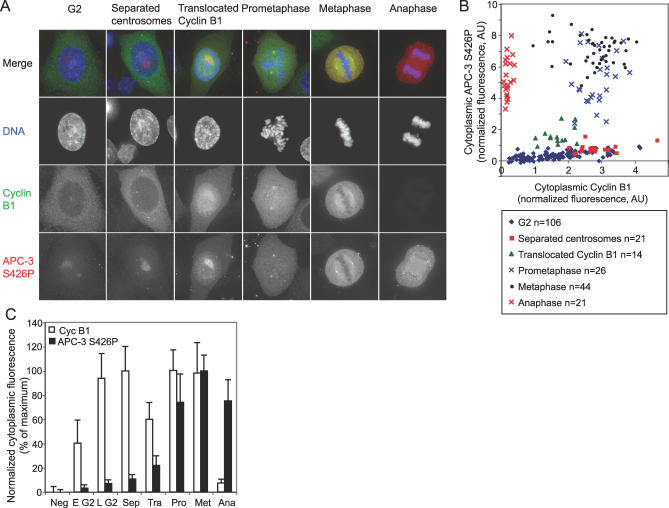
Gradual Phosphorylation of the Cdk1 Target APC3 S426 (A) Deconvolved maximum projections of unsynchronized HeLa cells. The cell-cycle stage is indicated above the figure. Top row: merge, second row: DNA, third row: cyclin B1, bottom row, APC3 S426P. (B) Dot plot of cytoplasmic cyclin B1 and cytoplasmic APC3 S426P staining. Each dot corresponds to one cell. Cells are arranged according to morphology and staining as in Figure 2A. (C) Average values of cytoplasmic cyclin B1 and phosphorylated APC3. Values are shown as percentage of the highest average. Error bars indicate SD. Bars are labeled as in Figure 2C.

### Cytoplasmic Cyclin B1–Cdk1 Dephosphorylation Continues after Nuclear Translocation of Cyclin B1

Subsequently, we focused on the development of cyclin B1–Cdk1 dephosphorylation when cyclin B1 enters the nucleus due to phosphorylation of its cytoplasmic retention signal in prophase [15,39]. It should be noted that the effect of nuclear translocation on development of cyclin B1–Cdk1 activity is puzzling. Translocation means a drop in the cytoplasmic cyclin B1 concentration, which could cause cytoplasmic Cdk1 activity to plummet. However, cytoplasmic cyclin B1–Cdk1 activity seems required to govern mitotic onset in the cytoplasm, e.g., to induce rearrangements such as microtubule polymerization and spindle assembly, involved in promoting nuclear envelope breakdown at the end of prophase [40,41]. In a bistable model, though, a sudden reduction of cyclin B1 from the cytoplasm at the end of prophase would not necessarily prevent cytoplasmic activation of cyclin B1–Cdk1 to proceed (see Text S1 and Figure S1).

When we compared cyclin B1 and phospho-Cdk1 levels before and after the onset of translocation (Figure 2A, red squares and green triangles), we observed that during cyclin B1 nuclear translocation, both the cytoplasmic cyclin B1– and Cdk1-P levels continued to decrease (Figures 1 and 2A; also see statistical analyses of the data in Figures 2C and S7). To further visualize the details of cyclin B1–Cdk1 dephosphorylation during nuclear translocation, we plotted the nuclear-to-cytoplasmic cyclin B1 ratio as a function of the ratio between cytoplasmic Cdk1-P and cyclin B1 in Figure 2B. When the ratio between phosphorylated Cdk1 and cyclin B1 was approaching 50% of that observed in early G2 cells, cyclin B1–Cdk1 translocated to the nucleus. Strikingly, in cells with translocated cyclin B1, the ratio between cytoplasmic Cdk1-P and cyclin B1 indeed continued to decrease, demonstrating the activation did proceed in the cytoplasm, too (Figure 2B). Thus, our results show that a pool of active cyclin B1–Cdk1 remains in the cytoplasm even throughout the nuclear translocation, potentially explaining how cyclin B1–Cdk1 activity can contribute to mitotic rearrangements taking place outside the nucleus at this time. Furthermore, these results indicate that once a positive feedback has been initiated, the activation of cyclin B1–Cdk1 is remarkably robust, and even proceeds upon a sudden drop in cyclin B1 concentration of approximately 50% (Figure 2C).

Total cyclin B1–Cdk1 activity in the cytoplasm is dependent on the concentration of the complex as well as on the fraction of active complexes present. We therefore reasoned that by multiplying the “relative activity” with the cyclin B1 levels, we would acquire a rough estimate of the “total cyclin B1–Cdk1 activity.” To estimate the relative activity, we assumed the ratio between P-Cdk1 and cyclin B1 in early G2 cells represented inactive complexes (Figures 2D and 2F). We then continued to plot the total cytoplasmic cyclin B1–Cdk1 activity as a function of cytoplasmic cyclin B1 levels, obtaining a loop representing activation and deactivation in G2 and mitosis (Figure 2D and 2E). In this representation, we see that the total cyclin B1–Cdk1 activity starts to increase in late G2, continues to rise to prometaphase, and remains high until cyclin B1 levels start to decline by inactivation of the spindle checkpoint and activation of Cdc20-APC/C in metaphase. The plot generated by our experimental data markedly resembled the relationship between in vitro Cdk1 activity and cyclin B concentration, observed in *Xenopus* egg extracts [9], with the important difference that human cytoplasmic cyclin B1 levels decreased as a result of nuclear translocation in prophase (Figure 2D, 2E, and 2F). Our results show that the principle of the cell-cycle oscillations between cyclin-Cdk activation and inactivation thus appears to be conserved in *Xenopus* and human cells.

### Phosphorylation of a Direct Cyclin B1–Cdk1 Target in Single Cells

Next, as an independent approach to determine cyclin B1–Cdk1 activity in human cells, we aimed to extend our analyses to a direct target of cyclin B1–Cdk1. As an additional benefit, this would allow us to confirm the kinetics of cyclin B1–Cdk1 activation when quantifying a positive readout signal. To this end, we used purified antibodies specifically recognizing phosphorylated APC3, a cyclin B1–Cdk1 substrate, in mitosis [42,43]. Figure S6A clearly shows the accumulation of APC3–Thr244-P on centrosomes after cyclin B1 translocation and during prometaphase, while the signal stabilized in metaphase and was greatly reduced in anaphase. Because cytoplasmic staining of this epitope was weak, next we analyzed the appearance of APC3–Thr446-P. Phosphorylation of this site was first identified in mitotic cells and has been reported to be induced by Cdk1, but not by Polo-like kinase 1, in vitro [44]. We saw the emergence of APC3–Thr446-P when centrosomes separated and cyclin B1 started to translocate to the nucleus in prophase (Figure S6B). For comparison, in the same manner as for Cdk1 dephosphorylation, we next quantitated the increase in APC3–Thr446-P exclusively in the cytoplasm. Figure S6C (gray bars) shows that cytoplasmic APC3–Thr446-P first rose slightly in G2 cells, slowly increasing after centrosome separation, and, interestingly, continued to accumulate while cyclin B1–Cdk1 translocated to the nucleus. Figure 3 shows a similar but more detailed quantification throughout G2 and mitosis of APC3–Ser426-P, a well-established Cdk1 target in vitro and in vivo, which is phosphorylated independently of Polo-like kinase 1 in vivo [44]. Accumulation of cytoplasmic APC3–Thr446-P and APC3–Ser426-P was highly comparable (Figure S6C). Cdk1-specific APC3 phosphorylation continued throughout prometaphase, indicating a need for enhanced cyclin B1–Cdk1 activity in prometaphase.

It should be noted that whereas the direct measurements in Figure 2 denote the activity of the cyclin B1–Cdk1 complex when the cell is fixed, the measurements in Figure 3 represent the accumulated sum of kinase as well as phosphatase activities. Moreover, APC3 is situated in both the nucleus and the cytoplasm, leading to an increased accessibility to cyclin B1–Cdk1 after nuclear envelope breakdown. Therefore, the detected APC3 phosphorylation lags slightly behind the detected Cdk1 dephosphorylation. However, both measurements showed a similar trend in gradually increasing cyclin B1–Cdk1 activity, in which the direct activity precedes the accumulated resulting phosphorylation.

### Fitting Activation to Mitotic Events: A Plot of Cyclin B1–Cdk1 Activation Kinetics

**Figure 4:**
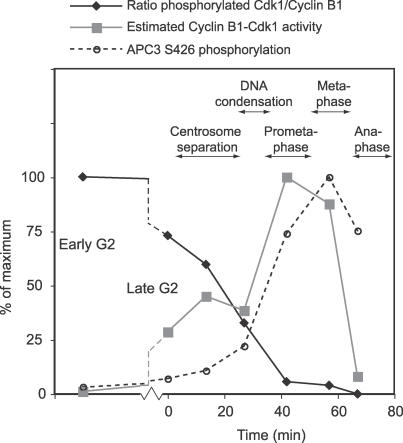
Schematic Representation of Cytoplasmic cyclin B1–Cdk1 Activation Average ratio of Cdk1-P and cyclin B1 staining (black solid line), average estimated cyclin B1–Cdk1 activity (gray solid line), and average APC-3 S426-P (black dotted line) plotted as a function of time (*x*-axis). The duration of mitotic events is derived from measurements described in Figure S8. Note the decrease in total cytoplasmic activity, but not in the progression of cyclin B1–Cdk1 dephosphorylation, between centrosome separation and nuclear translocation of cyclin B1–Cdk1.

To correlate the duration of Cdk1 activation with mitotic progression, we determined the timing of specific G2 and mitotic events: centrosome movement, DNA condensation, chromosome congression and sister chromatid separation in HeLa cells (Figure S8). In late G2, the centrosomes of most cells migrated from a position next to the nucleus to below the nucleus, where they separated. The activation of cyclin B1–Cdk1 preceded centrosome separation, but during the 27 min (SD = 13 min) between centrosome separation and DNA condensation, almost 50% of the cytoplasmic cyclin B1–Cdk1 complexes were activated (Figure 4, black line). At the same time, the amount of Ser426-phosphorylated APC3 started to increase (Figure 4, dotted line). Due to the nuclear translocation of cyclin B1–Cdk1, the total cytoplasmic activity was moderately decreased when DNA condensation became visible (Figure 4, gray line). We showed in Figure 2 that even during nuclear translocation, activation of cytoplasmic cyclin B1–Cdk1 complexes could continue. We also showed that accumulation of APC3–Ser426-P did not decrease during nuclear translocation. The chromosomes started to align on the metaphase plate about 20 min (SD = 9 min) after DNA condensation was initiated. During this process, cyclin B1–Cdk1 gained full activity, and as a consequence, the most dramatic increase in APC phosphorylation occurred.

In conclusion, cyclin B1–Cdk1 activity continued to accumulate between centrosome separation and chromosome congression in HeLa cells during a time span of approximately 45 min. The dephosphorylation of cyclin B1–Cdk1 proceeded in a gradual fashion, and the development of total cytoplasmic activity was modestly influenced by localization changes of the cyclin B1–Cdk1 complex (Figure 4).

### Implications for the Features of a Cdk1–APC/C Cell Cycle Oscillator

If bistability and hysteresis govern cyclin B1–Cdk1 activity, Cdk1 should not be phosphorylated and inactivated when the cyclin B1 concentration drops slightly below the activation threshold [45]. This occurs twice in an unperturbed cell cycle: during nuclear translocation of cyclin B1–Cdk1 in late G2, when cytoplasmic cyclin B1 levels decrease to the level of G2 cells in which cyclin B1–Cdk1 is inactive, and when cyclin B1 starts to be degraded in metaphase (Figures 1, 2D, 2E, and 2F). Indeed, we do not observe any increase of Cdk1-P during these events. Although we need to stress that this does not prove that mitosis is controlled by a bistable cyclin B1–Cdk1 circuit (since the system may not reach a steady state), we show that within the time-frame in which concentration changes occur in vivo, the activation curve for human cyclin B1–Cdk1 proceeds as if it were governed by bistability. It is probable that this contributes to the robustness and unidirectionality of mitosis, especially during the dramatic decrease in cytoplasmic cyclin B1–Cdk1 levels during nuclear translocation.

In a recent spatial theoretical simulation, Yang and coworkers [30] found that removing cyclin B1–Cdk1-dependent destruction of cyclin B1 turns the activation dynamics from limit cycle oscillations to bistability. Interestingly, in this model the cyclin B1–Cdk1 activity continues to rise in the cytoplasm during nuclear translocation. It should be noted that the negative feedback loop, leading to cyclin B1 inactivation, is not directly set off by the active state of cyclin B1–Cdk1. Rather, active cyclin B1–Cdk1 triggers mitotic entry, and the mitotic state is maintained by the spindle checkpoint, which prevents early activation of the APC/C-dependent negative feedback, even after the APC/C has already been phosphorylated by cyclin B1–Cdk1. This means that once cells are in the mitotic state with high cyclin B1–Cdk1 levels, they can remain in this state even when the negative feedback has been “preloaded” (i.e., the APC/C is maximally phosphorylated by cyclin B1–Cdk1). During a prolonged arrest, cyclin B1–Cdk1 activity remains high for hours in the checkpoint-arrested, mitotic state. Eventual slippage through the checkpoint is caused only by progressive, but very slow, degradation of cyclin B1 [46]. In normal mitotic cells, cyclin B1 is destroyed only when spindle checkpoint silencing releases inhibition of the APC/C, and Cdk1 rapidly returns to its low, interphase-state, activity.

### Effect on Mitotic Progression by Reducing Cyclin-Cdk1 Activity

Abrupt and efficient Cdk1 activation at the beginning of mitosis might be required to eventually trigger normal Cdk1 inactivation, which is necessary to build an uncompromised Cdk1-APC/C cell cycle oscillator in cycling extracts [9]. Rudner, Hardwick, and Murray reported that a slight decrease in specific Saccharomyces cerevisiae Cdk1 activity (by a mutation rendering active Cdc28 inefficient in ATP binding, or by mutation of the Cdc28 subunit Cks1) prevented Cdk-dependent hyperphosphorylation of the APC/C, resulting in stabilization of cyclins and Pds1 (S. cerevisiae Securin) and mitotic arrest [47]. These findings indicate that specific cyclin-Cdk1 kinase activity needs to accumulate during mitosis to prepare for efficient mitotic exit.

**Figure 5:**
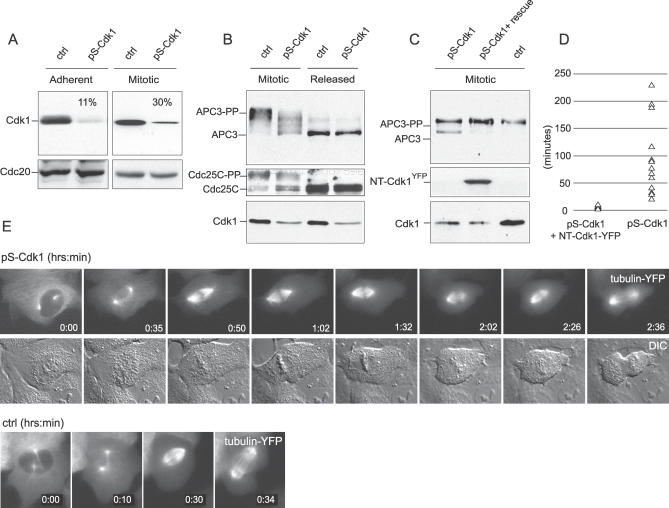
Distinct Requirement of Cdk1 for Mitotic Entry and Mitotic Progression in Human Cells (A) Cells selected for Cdk1 shRNA expression were synchronized in G2/M by thymidine release; mitotic cells were isolated by gentle shake-off. Mitotic cells were more than 95% MPM2 positive as analyzed by immunostaining and FACS analysis (unpublished data). Separated G2 and mitotic pools were analyzed for Cdk1 expression by Western blotting. Cdc20 protein levels serve as loading control. The percentage of remaining Cdk1 protein is indicated in the figure. (B) Cells collected by mitotic shake-off were lysed (lanes 1 and 2) or released from nocodazole and incubated in fresh medium for 3 h, recollected, and lysed (lanes 3 and4). Differences in mitotic phosphorylation shift of APC3 (human Cdc27 ortholog) and Cdc25C, depending on the Cdk1 levels, are shown (lanes 1 and 2). (C) The impaired phosphorylation of APC3 in Cdk1-attenuated mitotic cells (lane 1) was rescued by coexpression of a Cdk1-YFP construct containing a silent mutation in the RNAi targeting region (lane 2). Lane 3 are mitotic cells transfected with a control shRNA, revealing normal endogenous Cdk1 levels. (D) Distribution of metaphase duration, measured as time between chromosome alignment at the metaphase plate and onset of sister chromatid separation, in Cdk1 RNAi cells (right) or Cdk1 RNAi cells rescued by coexpression of non–RNAi-sensitive Cdk1-YFP (left). (E) Time-lapse microscopy analysis of mitotic progression after entry with normal or impaired Cdk1 levels. Bottom panels are consecutive images of tubulin-YFP in a pS-control cell in mitosis; top panels show delayed chromosome alignment (frames 2 and 3) and stalled metaphase (frames 4–6) after Cdk1 shRNA.

Next, to investigate the requirement for cyclin B1–Cdk1 at different phases of mitosis in human cells, we reduced the expression levels of Cdk1 by vector-driven shRNA (Figure 5A). As previously reported [48], we observed that reducing Cdk1 levels caused an increase in the population of G2 cells and a reduced fraction of cells entering mitosis after release from a G1/S block (unpublished data; see also Figure S9). However, approximately 20% of the cells with reduced Cdk1 levels initiated mitosis. When we compared the levels of Cdk1 in G2 and mitotic cells (collected by shake-off) after Cdk1 RNAi treatment, Cdk1 was selectively more abundant in the mitotic fraction (Figure 5A). This indicates that only cells with modestly reduced Cdk1 levels progressed from G2 to mitosis and suggests the existence of a threshold for Cdk1 at mitotic entry. These Cdk1-attenuated cells, which initiated mitosis, clearly showed reduced phosphorylation of the Cdk1 substrates APC3 and Cdc25C when arrested in nocodazole (Figure 5B), revealing that although the cyclin B1–Cdk1 activity was sufficient for entry into mitosis, phosphorylation of cyclin B1–Cdk1 targets remained incomplete. Coexpression of an RNAi-resistant Cdk1 construct together with the shRNA vector restored APC3 phosphorylation (Figure 5C), confirming the specificity of the RNAi. Biochemical analysis of cyclin B1–Cdk1 activity in cells that entered mitosis with reduced cyclin B1–Cdk1 levels revealed that the activity per complex remained largely unchanged, showing that the remaining cyclin B1–Cdk1 can still be activated even when total cyclin B1–Cdk1 levels are reduced, a result in agreement with a model of bistability (Figure S10). Release from nocodazole arrest revealed that Cdk1-attenuated cells retained the ability to exit from mitosis after 3–4 h (Figure 5B). However, when analyzed by time-lapse microscopy, cells that entered mitosis with reduced Cdk1 levels were strongly delayed before initiation of anaphase, with their chromosomes aligned at the metaphase plate (Figures 5D, 5E, and S9; average of 15 cells: 88 ± 65 min compared with 5 ± 3 min in 15 control cells, cotransfected with Cdk1 shRNA and RNAi-resistant Cdk1–yellow fluorescent protein [YFP]). Similar results were obtained in HeLa cells (unpublished data). Given the large number of Cdk1 substrates [49], it is likely these aberrancies can be attributed to impaired function of many cyclin B1–Cdk1 targets, which prohibited us from pinpointing a single mitotic process being affected. Nevertheless, taken together these results indicate that mitotic cyclin B1–Cdk1 activity needs to further increase after mitotic entry and pass a threshold before metaphase, necessary to allow activation of the machinery required for efficient mitotic progression and initiation of mitotic exit.

**Figure 6:**
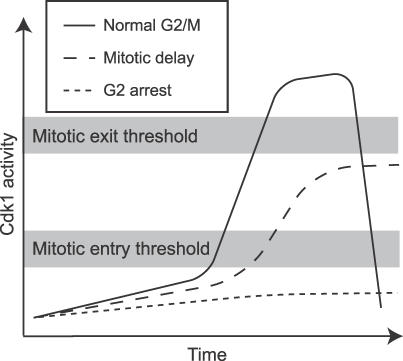
Different Cdk1 Activity Thresholds for Mitotic Entry and Mitotic Exit Model of relation between Cdk1 activity and mitotic progression. Cells do not enter mitosis unless a threshold concentration of active cyclin B1–Cdk1 is present (G_2_ arrest). After mitotic entry, the Cdk1 activity gradually increases, which enables the cell to prepare for mitotic exit (normal G2/M). If the Cdk1 activity does not develop fully after mitotic entry, the cell is delayed before anaphase (mitotic delay).

An important consequence of our findings is that distinct thresholds for cyclin B1–Cdk1 activity exist that control successive mitotic events. This concept has recently been demonstrated in mitotic exit, which requires the passage of different thresholds of decreasing cyclin B1–Cdk1 activity for the metaphase-to-anaphase and the anaphase-to-telophase transition [50,51]. Here, we propose a model where the cyclin B1–Cdk1 activity threshold for mitotic exit exceeds the threshold for mitotic entry, in line with observations in yeast [47] (Figure 6). It is tempting to speculate that the gradual activation of cyclin B1–Cdk1 during early mitosis, and the passage of distinct thresholds, critically assists in coordinating early and late mitotic events.

## Materials and Methods

### Cell culture, RNAi, and Western blots.

HeLa and U2OS cells were cultured in DMEM supplemented with Glutamax, 10% fetal calf serum, and antibiotics (GIBCO, http://www.invitrogen.com). Double-stranded oligos encoding shRNAs targeting human Cdk1 or cyclin B1 mRNA were cloned into the pSuper RNAi vector, under control of the H1 RNA promotor. Specificity and robustness of knock-down by various targeting vectors were tested by FACS analyses and Western blots using mouse anti-Cdk1 for detection, according to described procedures [52]. In this study, we used pSuper constructs driving short hairpins to target GGGGATTCAGAAATTGATC of human Cdk1 or a mixture of two plasmids to target GAACAGCTCTTGGGGACAT or GATGCTGCAGCTGGTTGGT of human cyclin B1. Specificity and stringency of the shRNA-induced gene knock-down were rigorously tested for the indicated RNAi experiments by Western blots, in comparison with unrelated shRNAs and rescue experiments of Cdk1 shRNA-induced phenotypes.

HeLa or U2OS cells were cotransfected using calcium phosphate and HBS with 5 or 10 μg of the indicated pSuper constructs, in the presence of 1 μg of α-tubulin–pEYFP as a reporter or 1 μg of pBABE-Puro as a selection marker, per 8-ml culture medium in 9-cm dishes of subconfluent cells or the equivalent for smaller dishes [52]. At 12 h after transfection, cells were selected with puromycin where indicated and synchronized in the presence of thymidine. Cells were released from thymidine block and puromycin selection after 20 h. Mitotic shake-offs were performed by a single gentle wash with warm PBS at 12 h after release from thymidine and selection or 14 h after release and 5 h after the addition of nocodazole (250 ng/ml). For shRNA-rescue experiments, a YFP-tagged cDNA construct of Cdk1 was generated harboring three silent mutations within the shRNA targeting regions and cotransfected with the Cdk1 shRNA vector at a 1:10 ratio. At 12 h after thymidine release, cell extracts were prepared using ELB buffer supplemented with protease and phosphatase inhibitors. Equalized protein samples were analyzed by Western blots using mouse anti–cyclin B1 (GNS1), mouse anti-Cdk1 (BD Transduction Laboratories, http://www.bdbiosciences.com), rabbit anti-Cdc20 (Santa Cruz Biotechnology, http://www.scbt.com), mouse anti-APC3 (BD Transduction Laboratories), or rabbit anti-Cdc25C (Santa Cruz).

### Time-lapse microscopy.

For three-dimensional time-lapse microscopy in Figure S8, see figure legend. For the analyses of mitotic progression after depletion of Cdk1, U2OS cells were plated onto glass-bottomed dishes (Willco Wells, http://www.willcowells.com) and transfected with 500 ng of pSuper-Cdk1 or control (either empty pSuper or pSuper targeting human Cks2, which is redundant in mitosis; R. M. F. Wolthuis, unpublished observations, and [53]), mixed with 40 ng of α-tubulin–YFP. Cells were transfected with indicated shRNAs and maintained in a climate-controlled culture chamber at the stage of a Zeiss Axiovert 200M microscope equipped with a 1.30 NA ×40 AxioPlan objective, specific dual band-pass filters, a 100-W xenon fast-shutter excitation device (DG4), and a Roper HQ Coolsnap CCD camera as shown previously [52]. Acquisition of DIC and fluorescence images started 40 h after transfection, at 100-ms exposure times. Images were captured and analyzed using MetaMorph and PhotoShop software.

### Immunofluorescence.

Cells were grown on hexametaphosphate/metasilicate-coated coverslips and fixed with PBS containing 3% paraformaldehyde and 2% sucrose for 10 min followed by permeabilization for 2 min in −20 °C methanol. Cells were incubated for 2 × 20 min in PBS containing 50 mM ammonium chloride before blocking in 2% bovine serum albumin and labeled with mouse anti–cyclin B1 (Santa Cruz, GNS1), rabbit anti-Y15P Cdk1 (Cdk1-P) (Cell Signaling, http://www.cellsignal.com), mouse anti-Cdk1 (Cell Signaling, POH-1); rabbit anti-phospho APC3-S426, rabbit anti-phospho APC3-S446 (kindly provided by Jan Michael Peters, IMP, Vienna, Austria), or rabbit anti-phospho APC3–Thr-244 (Abcam, http://www.abcam.com). After labeling with Alexa 488 anti-rabbit (Molecular Probes, http://www.probes.invitrogen.com), Cy3 anti-mouse (Jackson Immunoresearch, http://www.jacksonimmuno.com), and Hoechst 33342, the coverslip was mounted in Vectashield (Boehringer Mannheim, http://www.roche.com) or Mowiol 4–88 solution with antioxidants. z-stacks with 0.2-μm spacing were acquired using a Deltavision Spectris imaging system equipped with a ×60 objective, NA 1.4. A maximum intensity projection of the z-levels (SoftWorx; Applied Precision, http://www.api.com) is shown in Figure 1.

### Quantification of immunofluorescence.

Fifteen image z-stacks with 1-μm spacing were acquired in multiple locations of a single coverslip, using a Deltavision Spectris imaging system equipped with a ×20 objective, NA 0.7. To avoid bleaching of antibody fluorescence, cells were focused using the Hoechst labeling. The images were corrected for variation in illumination as determined by a photosensor (Applied Precision). For each z-stack, the background signal of an area without cells was subtracted. z-stacks where the background Cdk1-P signal differed more than 5% from the average background signal were not used. The average intensity of a square with five-pixel (3.3-μm) side was measured at the z-level that gave the highest average intensity in the cytoplasm, at a place where no obvious structures (e.g., centrosome, spindle, strong dotted staining) were present, of 318 cyclin B1–expressing cells using SoftWorx (Applied Precision). For examples of quantification, see Figure S3. The signal from prometaphase, metaphase, and anaphase cells was corrected for morphological difference by division of the signal by 1.38, as assessed by the difference in cytoplasmic NF-κB staining between G2 and mitotic cells Figure S4. The average background signal of cells not expressing cyclin B1 was quantified and subtracted from the measurements, with morphology corrections for prometaphase, metaphase, and anaphase cells.

## Supporting Information

Figure S1 The Concepts of Bistability and Limit Cycle Oscillations for Cyclin B1–Cdk1 Activation For details on the figure, see text S1.
(89 KB PDF)

Figure S2 Specificity of Cdk1-P and Cyclin B1 Antibodies in Immunofluorescence HeLa cells were microinjected with 0.005 μg/μl pCFP-Golgi together with 0.1 μg/μl pS-Cdk1 or pS-cyclin B1, synchronized by a single 24-h thymidine block and fixed 10–20 h after release. Labeling was performed as in Figure 1, with the addition of B1:5 guinea pig anti-Cdc25B [54].
(A) Undeconvolved maximum intensity projections of immunofluorescence staining of HeLa cells. Cells with separated centrosomes and high Cdc25B levels, indicating late G2 phase, are shown. Top, uninjected cell. Middle, cell injected with pSuper–cyclin B1 and pCFP-Golgi. Bottom, cell injected with pSuper-Cdk1 and pCFP-Golgi. All cells were growing on the same coverslip.
(B) Colocalization of the cytoplasmic Cdk1-P and cyclin B1 stainings of top cell in Figure S2A. Colocalization was assessed with ImageJ using the plugin Colocalization_finder. Pixels situated inside mask, where the relation between the two stainings is linear, are indicated in white. The Cdk1-P and cyclin B1 stainings thus colocalize linearly in the cytoplasm, whereas although they colocalize also in the nucleus and on the centrosomes, part of the staining there comes from unspecific signal.
(2.3 MB PDF)

Figure S3 Examples of Quantification of the Cdk1-P and Cyclin B1 Stainings Stacks of DNA, cyclin B1, and Cdk1-P stainings were acquired on multiple locations on a single coverslip. The average intensity of a square with 3.3-μm side was measured in the cytoplasm at the z-level that gave the highest cyclin B1 readout. The square was placed so that visible structures (centrosomes, mitotic spindle, strong dotted patterns, or dots that may come from secondary antibody precipitates) in both the cyclin B1 and Cdk1-P stainings were not included. Top row. G2 cell. Middle row, late prometaphase cell. Bottom row, cell with translocated cyclin B1. Note that the middle row measurements are made at a different z-level (as indicated by the z-marker in the lower left corner of each image).
(9.3 MB PDF)

Figure S4 Correction of Quantifications for Morphology Changes during Mitosis (A) The NF-κB staining is cytoplasmic in untreated HeLa cells. Images are undeconvolved maximum intensity projections of NF-κB, cyclin B1, and DNA stainings.
(B) Immunofluorescence quantifications are slightly overestimated in mitotic cells. The cytoplasmic NF-κB signal was measured at similar settings as for the quantifications of cyclin B1 and phosphorylated Cdk1, as shown in Figure S3. Bars show the average value of the cytoplasmic NF-κB signal in cyclin B1–positive (G2) and mitotic cells. Error bars indicate standard deviation. The acquired ratio (1.38) was used to correct the signal of mitotic cells in all measurements in this article.
(1.0 MB PDF)

Figure S5 Cdk1 Levels Do Not Decrease during Mitosis (A) Cdk1 levels do not decrease during mitosis. Images show unsynchronized HeLa cells stained for cyclin B1, Cdk1, and DNA.
(B) Quantification of total Cdk1 immunofluorescence labeling. Cytoplasmic Cdk1 (*y*-axis) and cyclin B1 signal (*x*-axis) was quantified in the cytoplasm of unsynchronized cells. Each dot corresponds to one cell. Cells are labeled according to morphology and staining as follows: G2 cells (blue diamonds), mitotic cells before anaphase (red squares), anaphase cells (green triangles).
(643 KB PDF)

Figure S6 Characterization of APC3 T244P and APC3 S446P Antibodies throughout Mitosis (A) APC3 T244 is mainly present on the centrosomes. Images are maximum intensity projections of deconvolved immunofluorescence stainings.
(B) APC3 S446 is phosphorylated both in the nucleus and in the cytoplasm. Images are maximum intensity projections of deconvolved immunofluorescence stainings.
(C) APC3 S426 and APC3 T446 show similar cytoplasmic phosphorylation patterns. The levels of APC3–S426-P (Figure 3C) and the estimated cyclin B1–Cdk1 activity (Figure 2F) are included for comparison. Values are shown as percentage of the highest average. Error bars indicate standard deviation. Neg, cells used for background subtraction; E G2, G2 cells with cyclin B1 levels between average anaphase levels and lowest value of cell with separated centrosomes; L G2, G2 cells with cyclin B1 levels above lowest value of cell with separated centrosomes; Sep, separated centrosomes; Tra: translocated cyclin B1; Pro, prometaphase; Met, metaphase; Ana, anaphase.
(6.4 MB PDF)

Figure S7 Statistical Analysis of Quantifications in Figures 2F and 3C The clamp between two bars indicates a statistical difference (*p* < 0.05) between the samples. The statistical analysis was performed using the formula:
X_1_ − X_0_ ± 1.96 × √(S_1_^2^/n_1_ + S_0_^2^/n_2_)
(A) Cytoplasmic ratio of P-Cdk1 and cyclin B1, from Figure 2F.
(B) Cytoplasmic APC3 S426P
(491 KB PDF)

Figure S8 Time-frame of Some Mitotic Events in HeLa Cells HeLa cells growing on a glass-bottom dish (MatTek) were microinjected with 0.008 μg/μl YFP-Histone H2B (green) and 0.0016 μg/μl dsRED-γ-tubulin (red). After a 24-h thymidine block, cells were moved to a Deltavision Spectris microscope (Applied Precision) equipped with a heater and CO_2_ chamber (Solent Scientific). Using a ×60 objective, NA 1.4, 15 z-levels with 1-μm spacing were acquired on multiple locations in the dish every 12 min. The distance between centrosomes was measured using SoftWorx (Applied Precision). For figures, a maximum intensity projection was made of the z-levels.
(A) Maximum intensity projections of a cell entering mitosis. The centrosomes are indicated with arrows.
(B) The average time in minutes, with SD in brackets, between early mitotic events. Time is counted from the first image where the indicated event is observed. See text for details.
(1.9 MB PDF)

Figure S9 Effects of Cdk1 Attenuation on Mitotic Progression (A) Details of early mitosis revealing delayed spindle formation in cells entering mitosis with reduced Cdk1 levels.
(B) A cell arresting at the G2-M transition after centrosome separation. Mitotic entry after centrosome separation usually occurs within approximately 10–20 min in U2OS cells, e.g., see Figure 5D.
(7.0 MB PDF)

Figure S10 Cyclin B1–Cdk1 Complexes from pSuper- or pSuper-Cdk1–Treated Cells Are Comparably Active (A) Mitotic U2OS cells were collected after treatment with either pSuper or pSuper-Cdk1. Extracts of these cells were blotted with indicated antibodies. Importantly, next to a reduction of Cdk1 levels, cyclin B1 levels also were reduced. This indicates that only around 40% of cyclin B1–Cdk1 complexes could be formed in pSuper-Cdk1–treated cells compared to control cells.
(B and C) Cell extracts from (A) were subjected to immunoprecipitation with cyclin B1 antibodies for 4 h at 4 °C. Next, the cyclin B1 immunoprecipitates were incubated with ^32^P-γATP for 30 min with Histon H1 as substrate. We separated different amounts of the reactions on gel to correct for possible differences in cyclin B1 and/or Cdk1 levels in the IPs. Western blots were performed using cyclin B1 and Cdk1 antibodies after exposures were taken using PhosphorImager films. Intensities were measured and background was subtracted using MetaMorph software and Excel. The highest signal in the pSuper samples was set at 100%, and the estimated kinase activity for every lane was plotted against protein levels of cyclin B1 (B) or Cdk1 (C) present in the same lane. In both mitotic samples, the kinase activity roughly followed the same line. Only the activity per cyclin B1 molecule from the pSuper-Cdk1 cells seemed slightly lower (B), which can be explained if cyclin B1 exceeds the remaining amount of Cdk1. Indeed, correlating kinase activity with the Cdk1 levels revealed a closer overlap (C). All together, these data show that pS-Cdk1 cells have fewer cyclin B1–Cdk1 complexes but the complexes that are formed are fully active.
(1.2 MB PDF)

Text S1 Small Glossary of Nonlinear Dynamics in the Cell Cycle (21 KB DOC)

## Abbreviations

APC/C: anaphase-promoting complex/cyclosome
Cdk: cyclin-dependent kinase
NF-κB: nuclear factor κB
P: phosphorylation
RNAi: RNA interference
shRNA: short hairpin RNA
YFP: yellow fluorescent protein
