# Supplementary material for: Cyclin B1–Cdk1 Activation Continues after Centrosome Separation to Control Mitotic Progression
Source: PLoS Biol. 2007 May 1;5(5):e123. doi: 10.1371/journal.pbio.0050123 (PMC1858714; doi:10.1371/journal.pbio.0050123)
Supplement: Supplementary file 1 [file pbio.0050123.sg001.pdf]

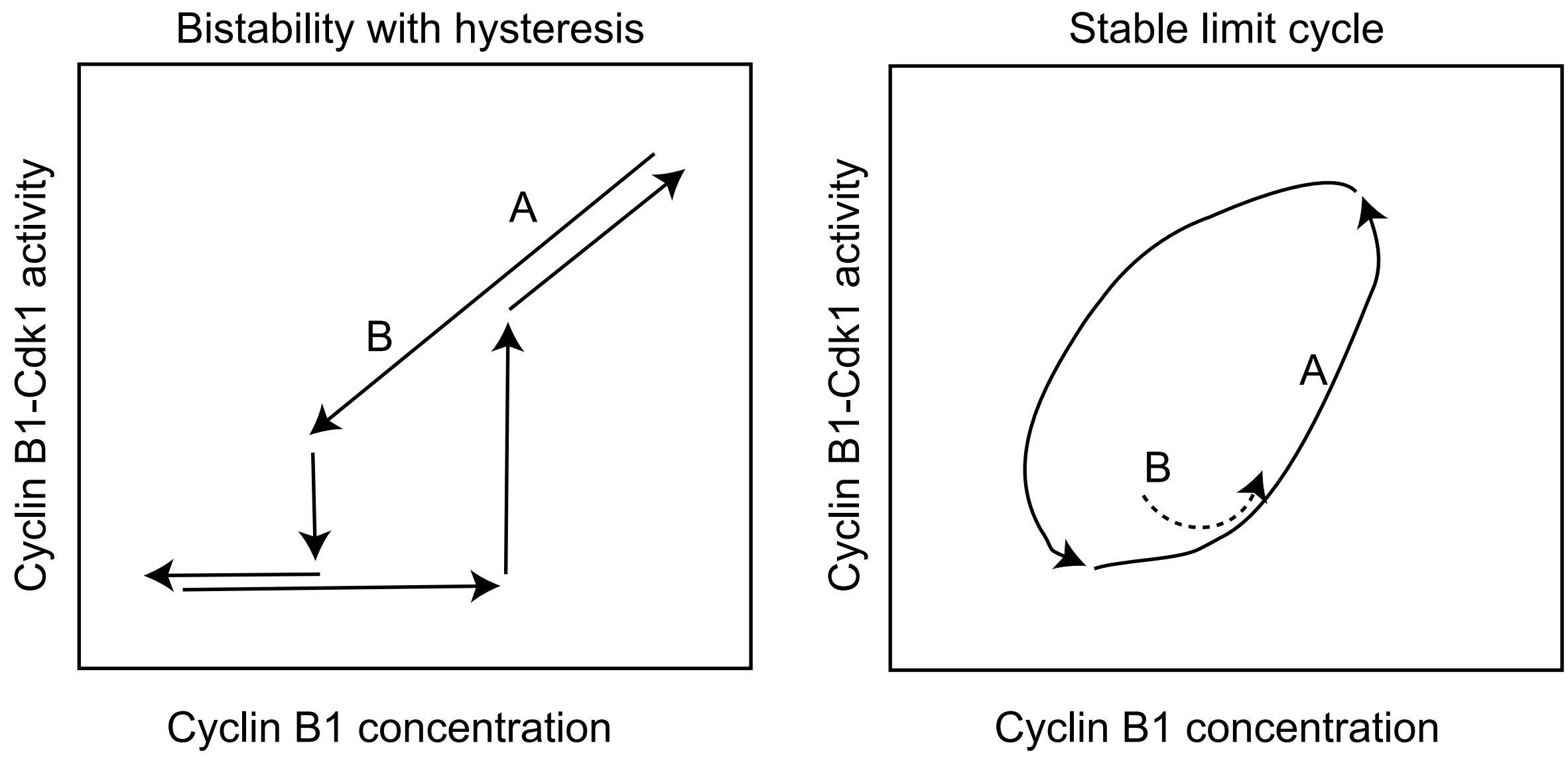
Bistability with hysteresis
Cyclin B1-Cdk1 activity
A
B
Cyclin B1 concentration
Stable limit cycle
Cyclin B1-Cdk1 activity
A
B
Cyclin B1 concentration

Figure S1
