# Supplementary material for: Cyclin B1–Cdk1 Activation Continues after Centrosome Separation to Control Mitotic Progression
Source: PLoS Biol. 2007 May 1;5(5):e123. doi: 10.1371/journal.pbio.0050123 (PMC1858714; doi:10.1371/journal.pbio.0050123)
Supplement: Supplementary file 2 [file pbio.0050123.sg002.pdf]

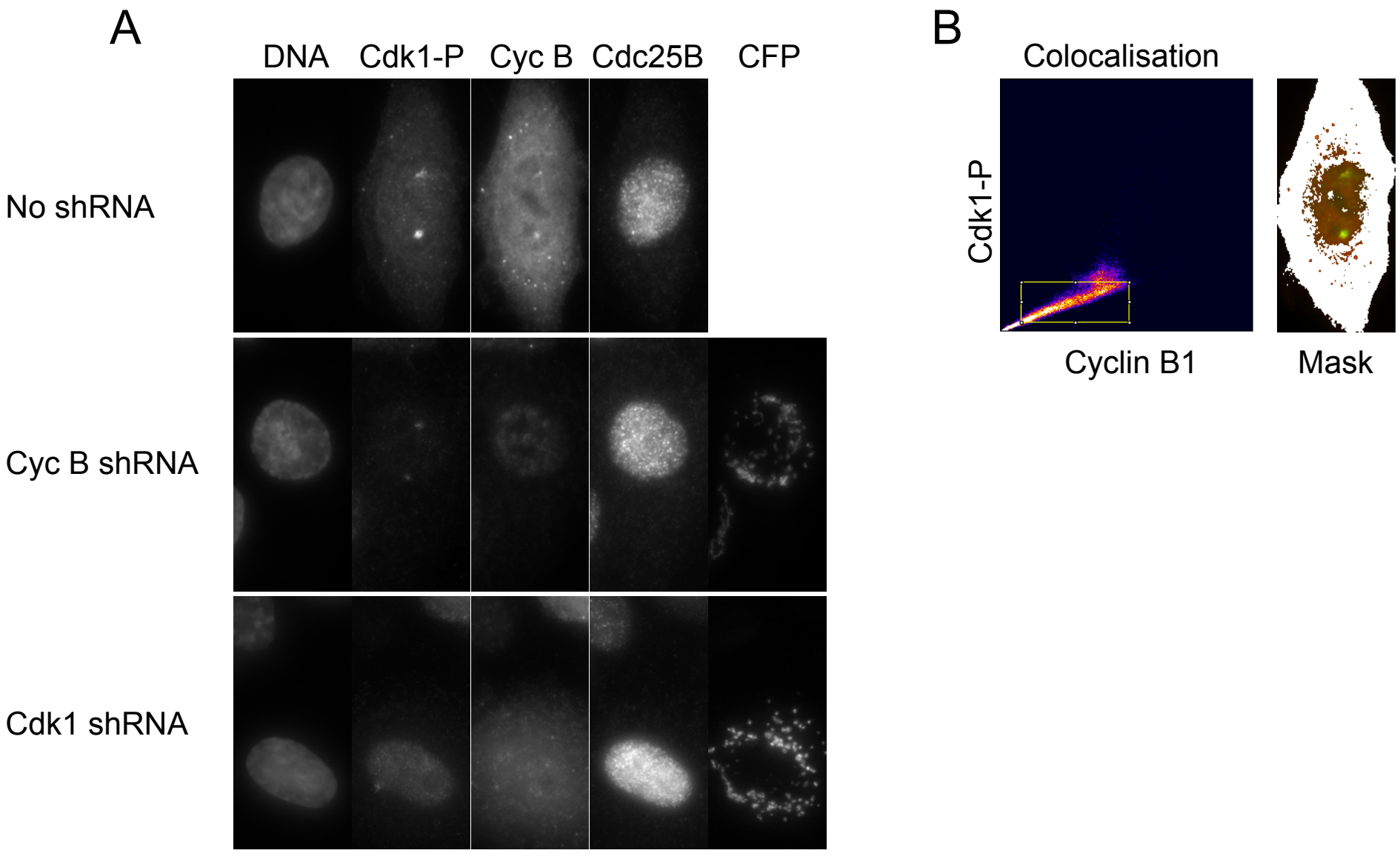
A
DNA
Cdk1-P
Cyc B
Cdc25B
CFP
No shRNA
Cyc B shRNA
Cdk1 shRNA
B
Colocalisation
Cdk1-P
Cyclin B1
Mask

Figure S2
