# Supplementary material for: Cyclin B1–Cdk1 Activation Continues after Centrosome Separation to Control Mitotic Progression
Source: PLoS Biol. 2007 May 1;5(5):e123. doi: 10.1371/journal.pbio.0050123 (PMC1858714; doi:10.1371/journal.pbio.0050123)
Supplement: Supplementary file 3 [file pbio.0050123.sg003.pdf]

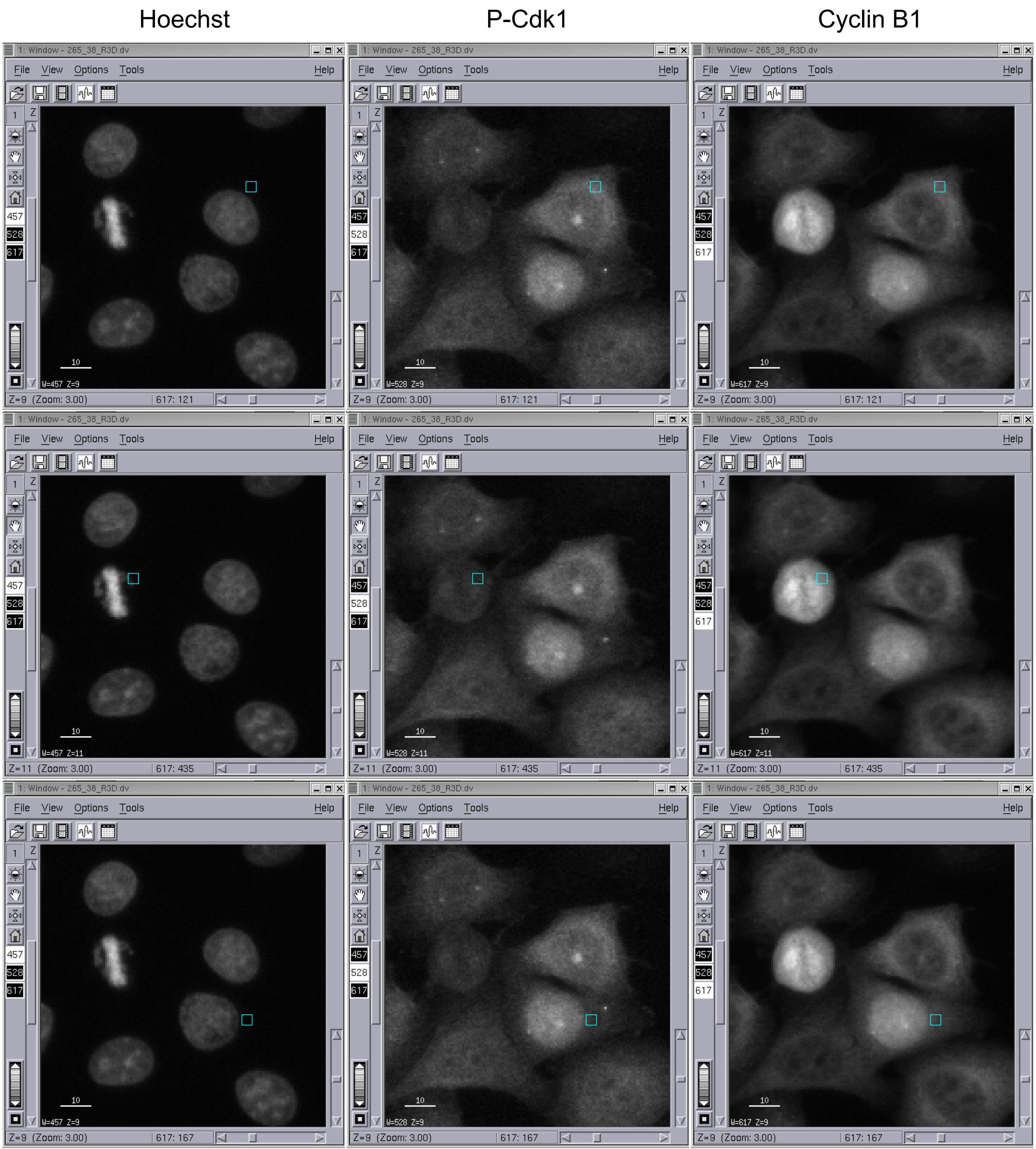

Figure S3
