# Supplementary material for: Cyclin B1–Cdk1 Activation Continues after Centrosome Separation to Control Mitotic Progression
Source: PLoS Biol. 2007 May 1;5(5):e123. doi: 10.1371/journal.pbio.0050123 (PMC1858714; doi:10.1371/journal.pbio.0050123)
Supplement: Supplementary file 5 [file pbio.0050123.sg005.pdf]

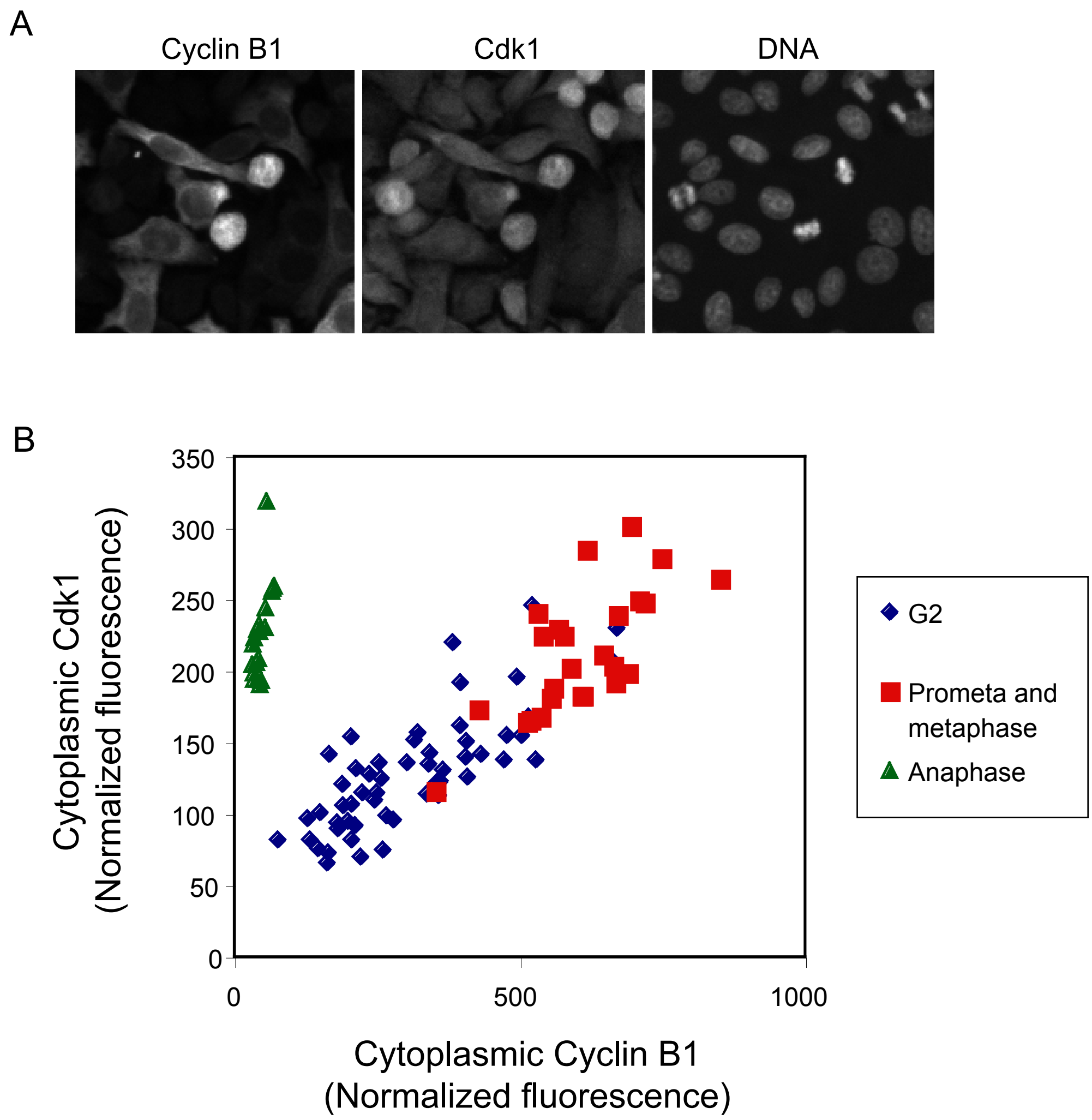
A
Cyclin B1
Cdk1
DNA
B
Cytoplasmic Cdk1
(Normalized fluorescence)
350
300
250
200
150
100
50
0
0
500
1000
Cytoplasmic Cyclin B1
(Normalized fluorescence)
G2
Prometa and metaphase
Anaphase

Figure S5
