# Supplementary material for: Cyclin B1–Cdk1 Activation Continues after Centrosome Separation to Control Mitotic Progression
Source: PLoS Biol. 2007 May 1;5(5):e123. doi: 10.1371/journal.pbio.0050123 (PMC1858714; doi:10.1371/journal.pbio.0050123)
Supplement: Supplementary file 6 [file pbio.0050123.sg006.pdf]

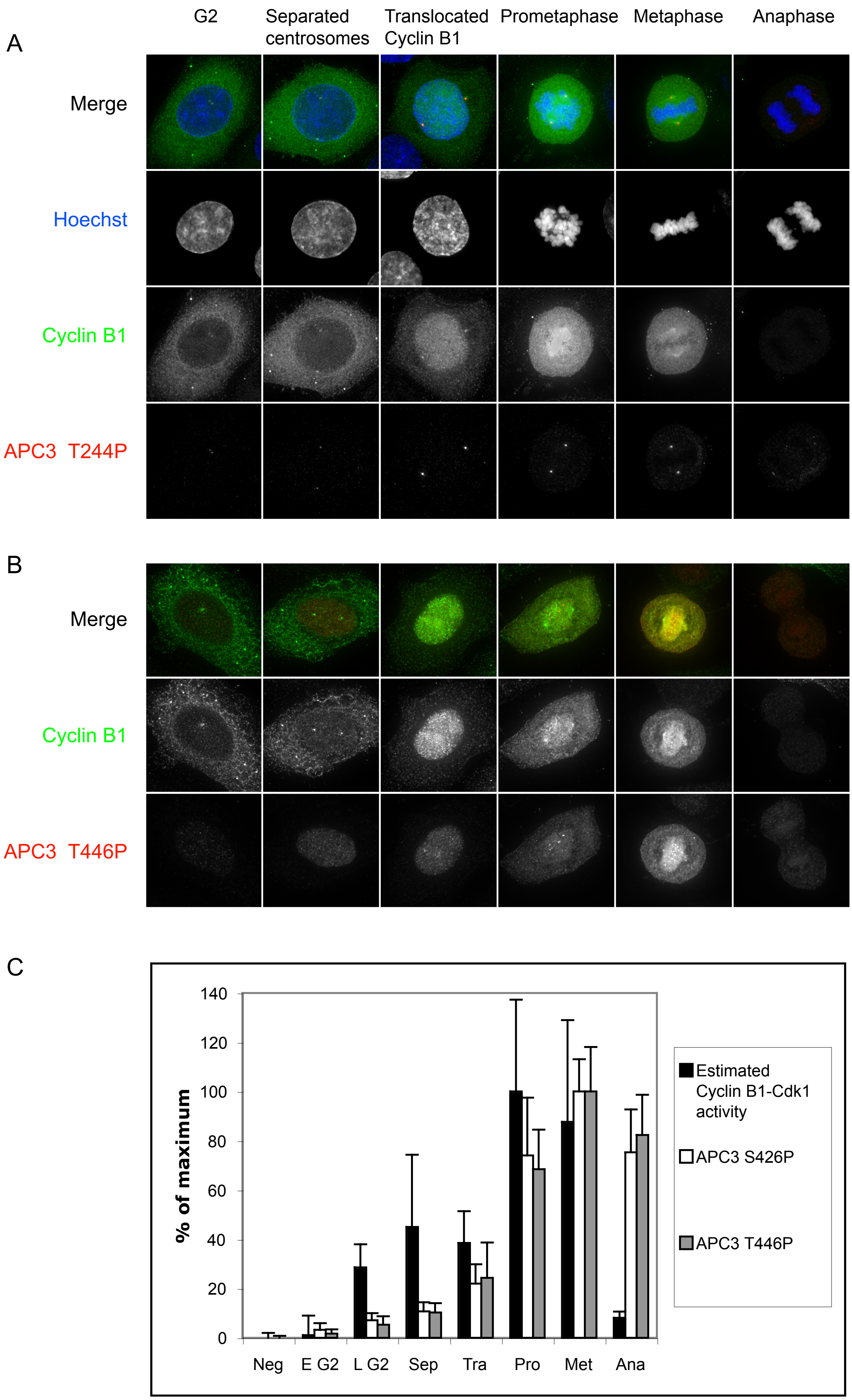
A
G2
Separated centrosomes
Translocated Cyclin B1
Prometaphase
Metaphase
Anaphase
Merge
Hoechst
Cyclin B1
APC3 T244P
B
Merge
Cyclin B1
APC3 T446P
C
% of maximum
140
120
100
80
60
40
20
0
Neg
E G2
L G2
Sep
Tra
Pro
Met
Ana
Estimated Cyclin B1-Cdk1 activity
APC3 S426P
APC3 T446P

Figure S6
