# Supplementary material for: Cyclin B1–Cdk1 Activation Continues after Centrosome Separation to Control Mitotic Progression
Source: PLoS Biol. 2007 May 1;5(5):e123. doi: 10.1371/journal.pbio.0050123 (PMC1858714; doi:10.1371/journal.pbio.0050123)
Supplement: Supplementary file 7 [file pbio.0050123.sg007.pdf]

A

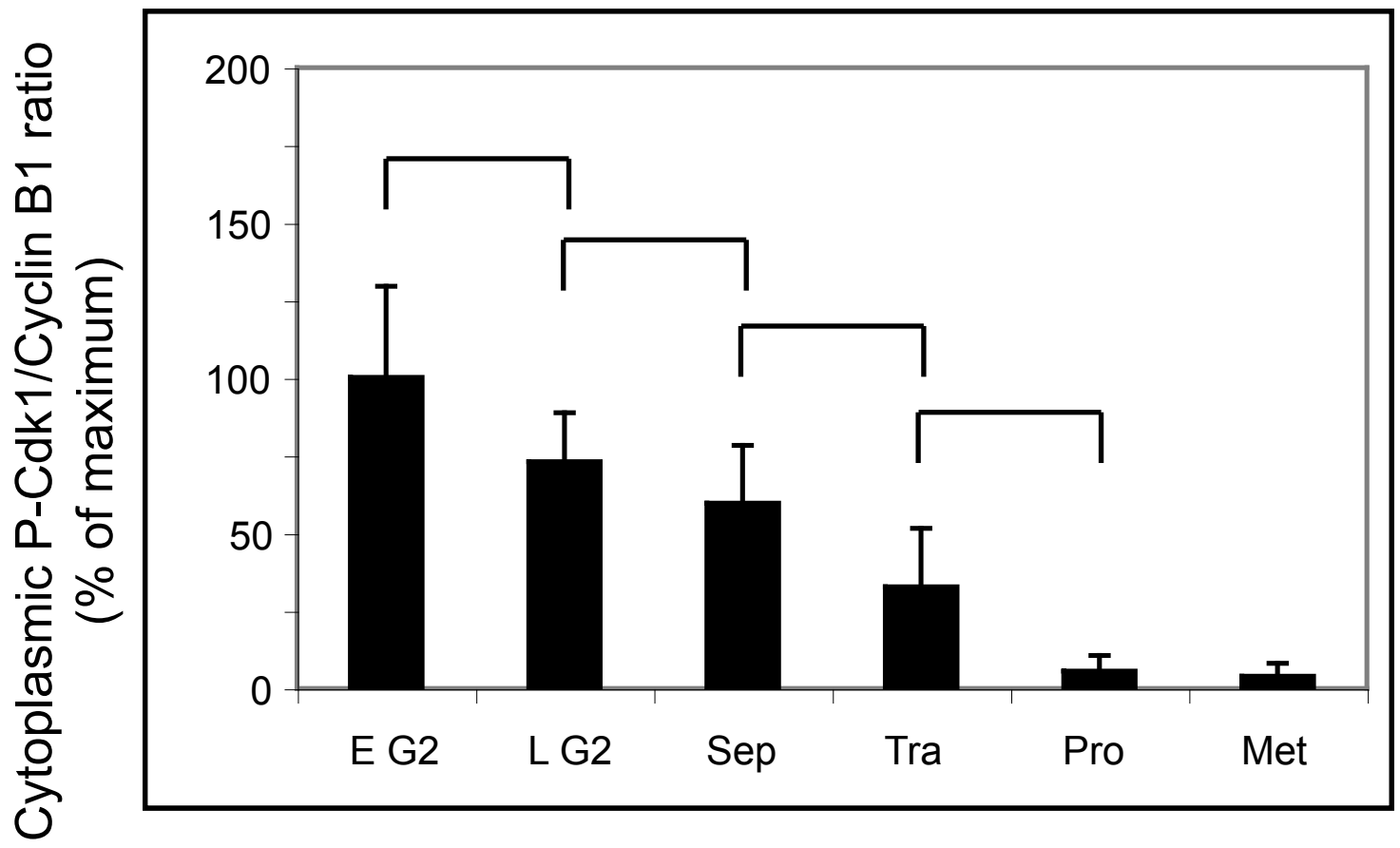
Cytoplasmic P-Cdk1/Cyclin B1 ratio
(% of maximum)
200
150
100
50
0
E G2
L G2
Sep
Tra
Pro
Met

B

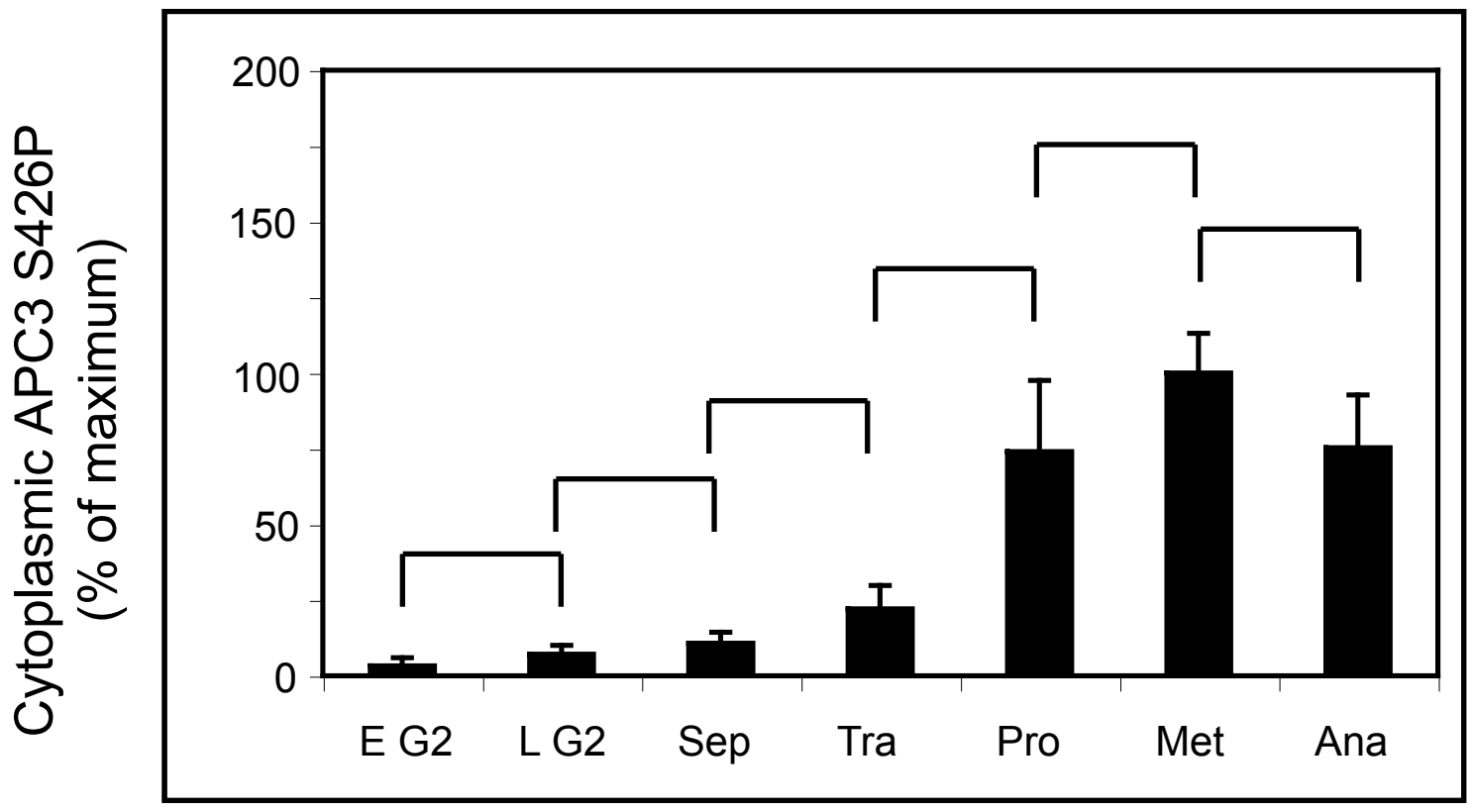
Cytoplasmic APC3 S426P
(% of maximum)
200
150
100
50
0
E G2
L G2
Sep
Tra
Pro
Met
Ana

Figure S7
