# Supplementary material for: Cyclin B1–Cdk1 Activation Continues after Centrosome Separation to Control Mitotic Progression
Source: PLoS Biol. 2007 May 1;5(5):e123. doi: 10.1371/journal.pbio.0050123 (PMC1858714; doi:10.1371/journal.pbio.0050123)
Supplement: Supplementary file 8 [file pbio.0050123.sg008.pdf]

A

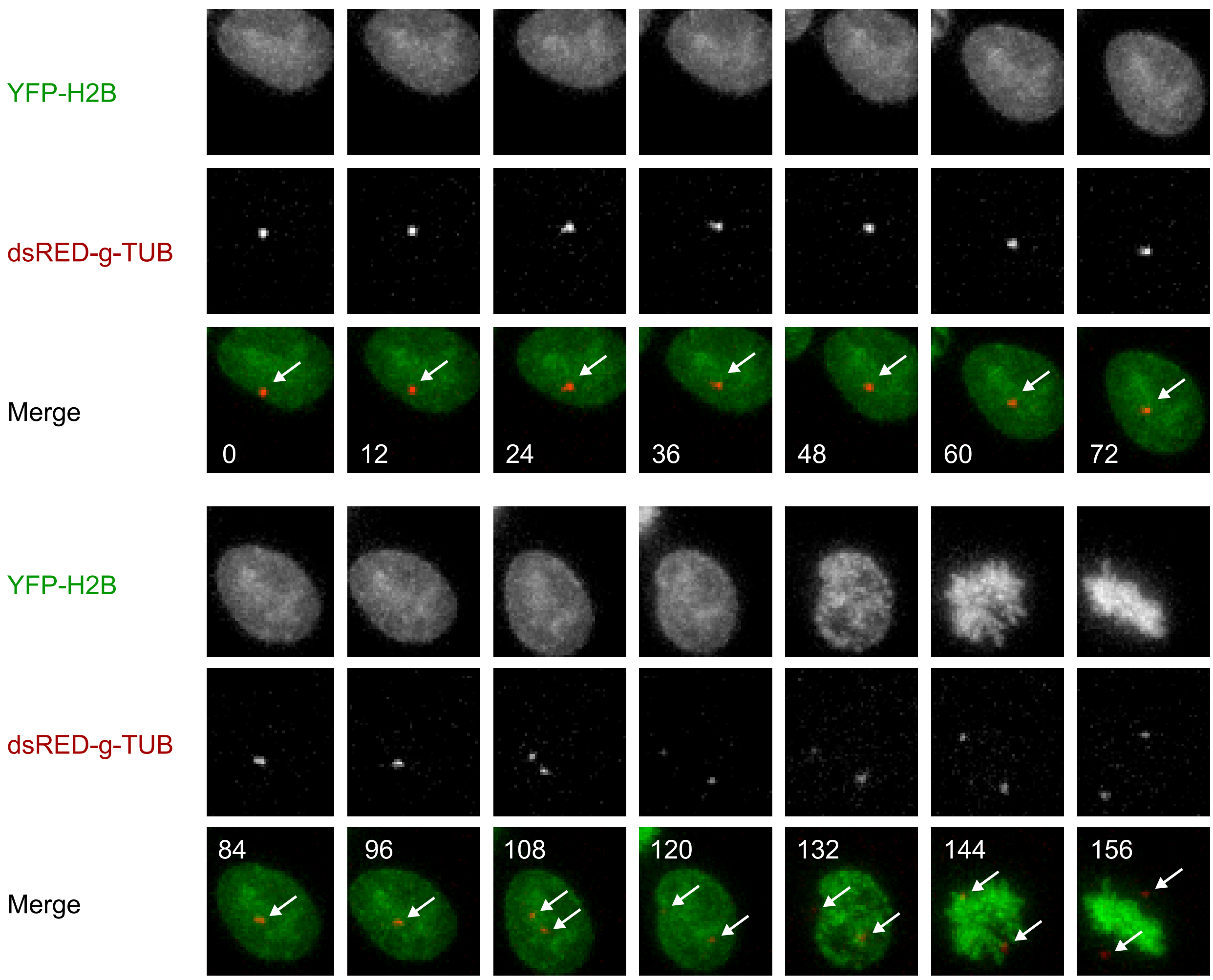

B

Centrosome separation	
	27 (13)
DNA condensation	
	20 (9)
Chromosome congression	
	31 (16)
Anaphase	

Figure S8
