# Supplementary material for: Cyclin B1–Cdk1 Activation Continues after Centrosome Separation to Control Mitotic Progression
Source: PLoS Biol. 2007 May 1;5(5):e123. doi: 10.1371/journal.pbio.0050123 (PMC1858714; doi:10.1371/journal.pbio.0050123)
Supplement: Supplementary file 9 [file pbio.0050123.sg009.pdf]

A

α-tubulin -YFP

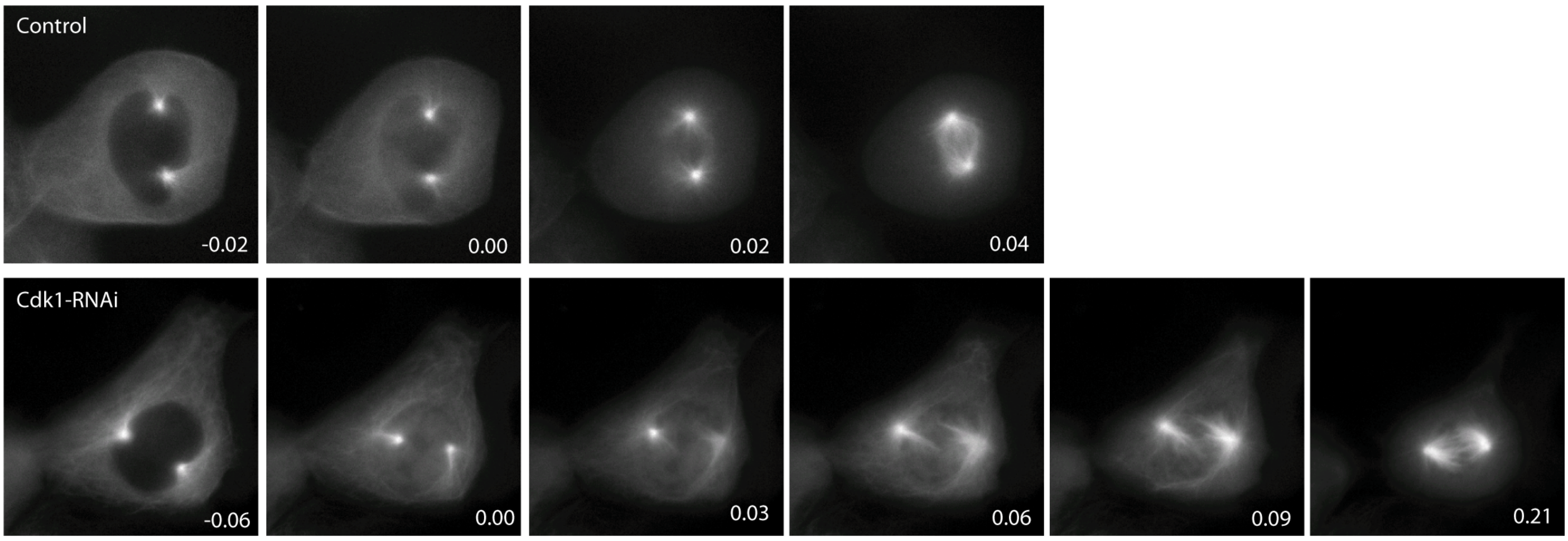

B

α-tubulin -YFP + Cdk1 RNAi

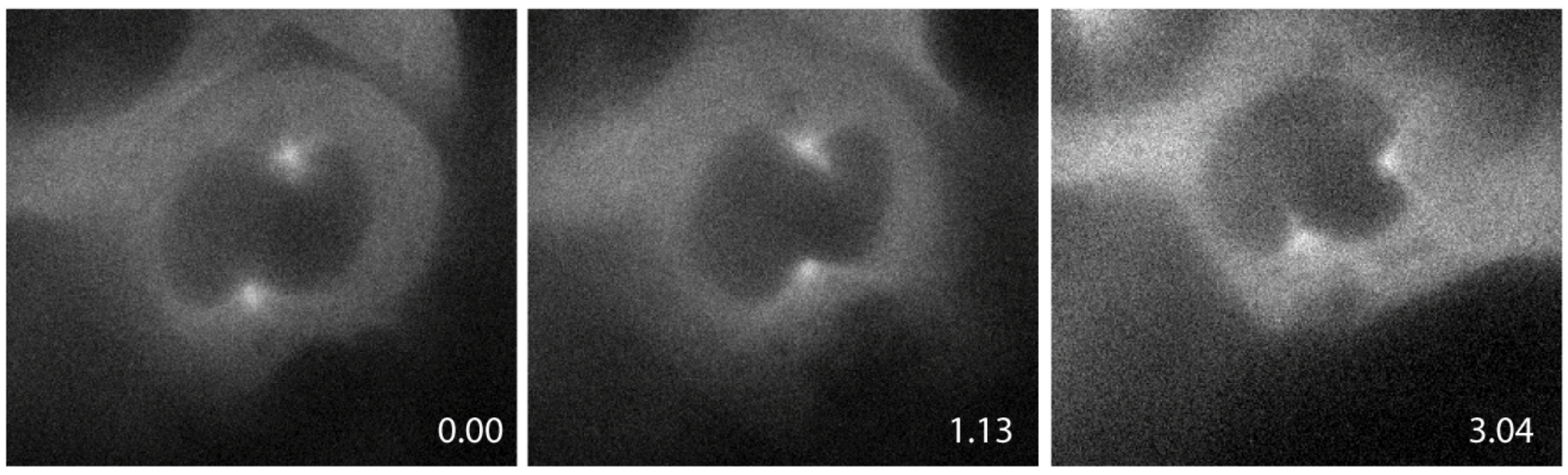

Figure S9
