# Supplementary material for: Cyclin B1–Cdk1 Activation Continues after Centrosome Separation to Control Mitotic Progression
Source: PLoS Biol. 2007 May 1;5(5):e123. doi: 10.1371/journal.pbio.0050123 (PMC1858714; doi:10.1371/journal.pbio.0050123)
Supplement: Supplementary file 10 [file pbio.0050123.sg010.pdf]

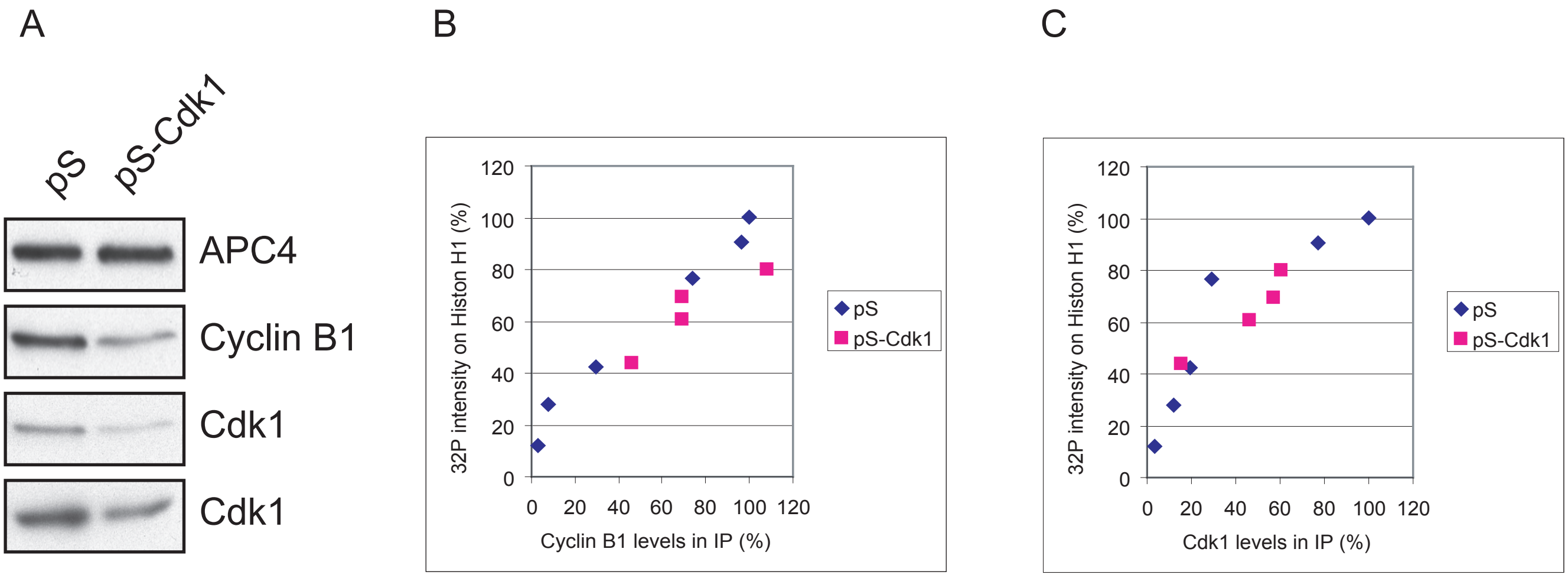
A
pS
pS-Cdk1
APC4
Cyclin B1
Cdk1
Cdk1
B
120
100
80
60
40
20
0
32P intensity on Histon H1 (%)
0 20 40 60 80 100 120
Cyclin B1 levels in IP (%)
pS
pS-Cdk1
C
120
100
80
60
40
20
0
32P intensity on Histon H1 (%)
0 20 40 60 80 100 120
Cdk1 levels in IP (%)
pS
pS-Cdk1

Figure S10
