# Supplementary material for: Cyclin B1–Cdk1 Activation Continues after Centrosome Separation to Control Mitotic Progression
Source: PLoS Biol. 2007 May 1;5(5):e123. doi: 10.1371/journal.pbio.0050123 (PMC1858714; doi:10.1371/journal.pbio.0050123)
Supplement: Supplementary file 11 [file pbio.0050123.sd001.doc]

**Supporting Text.**

**Small Glossary of Non-linear Dynamics in the Cell Cycle**

In the cell cycle, activation of Cyclin B1-Cdk1 is the target of positively and negatively interfering pathways that control the commitment of G2 cells to enter mitosis. Once started, mitosis is irreversible, only leading either to correct cell division or to genomically instable G1 cells prone to cell death. It is therefore critical to understand how Cyclin B1-Cdk1 can switch from an inactive to an active state. A network of signal transduction events, involving multiple feedback loops, can show several characteristics that are not easily foreseen intuitively. From theoretical models on signal transduction pathways, known to regulate Cyclin B1-Cdk1 activity, two main concepts have evolved: bistability and limit cycle oscillations.

**Bistability** means that a system can rest in two stable states. If the system is pushed to any other state, it will respond by progressing towards one of the stable states. An inherent property of bistable systems is the dependence of the systems history: a bistable system being pushed to the middle between two stable states will return to the stable state it was coming from. The conservative delay in switching to a novel state is referred to as **hysteresis**. In case of Cyclin B1-Cdk1, this would mean its activity can remain high even in case the Cyclin B1-Cdk1 levels temporally decrease below the levels for an activation threshold (from point A to point B in the figure). Note that the figure denotes endpoints and thus does not include information on how long the activation takes.

**Limit cycle oscillations** occur when a system cannot rest in a stable state. Instead the system constantly changes, thus creating oscillations. A stable limit cycle will oscillate along a closed trajectory. If the stable limit cycle oscillator is pushed (to many points) outside this trajectory, it will approach the original trajectory over time. If Cyclin B1-Cdk1 activity follows a stable limit cycle, interfering with Cyclin B1 levels during increasing activity would temporally change the trajectory of the activation. In case such a reduction of Cyclin B1 levels is large enough (from point A to point B in figure S1), a temporal decrease in Cyclin B1-Cdk1 activation will occur.

In the text, we argue that the change from point A to point B in figure S1 can be compared to the situation in the cytoplasm during nuclear translocation of Cyclin B1-Cdk1 in prophase.
